# Functional characterization of a second pedal peptide/orcokinin‐type neuropeptide signaling system in the starfish *Asterias rubens*

**DOI:** 10.1002/cne.24371

**Published:** 2017-12-18

**Authors:** Ming Lin, Michaela Egertová, Cleidiane G. Zampronio, Alexandra M. Jones, Maurice R. Elphick

**Affiliations:** ^1^ School of Biological & Chemical Sciences, Mile End Road Queen Mary University of London London United Kingdom; ^2^ School of Life Sciences and Proteomics Research Technology Platform University of Warwick Coventry United Kingdom

**Keywords:** deuterostome, echinoderm, neuropeptide, orcokinin, pedal peptide, starfish, RRID: AB_2716765

## Abstract

Molluscan pedal peptides (PPs) and arthropod orcokinins (OKs) are prototypes of a family of neuropeptides that have been identified in several phyla. Recently, starfish myorelaxant peptide (SMP) was identified as a PP/OK‐type neuropeptide in the starfish *Patiria pectinifera* (phylum Echinodermata). Furthermore, analysis of transcriptome sequence data from the starfish *Asterias rubens* revealed two PP/OK‐type precursors: an SMP‐type precursor (*A. rubens* PP‐like neuropeptide precursor 1; ArPPLNP1) and a second precursor (ArPPLNP2). We reported previously a detailed analysis of ArPPLNP1 expression in *A. rubens* and here we report the first functional characterization ArPPLNP2‐derived neuropeptides. Sequencing of a cDNA encoding ArPPLNP2 revealed that it comprises eleven related neuropeptides (ArPPLN2a‐k), the structures of several of which were confirmed using mass spectrometry. Analysis of the expression of ArPPLNP2 and neuropeptides derived from this precursor using mRNA in situ hybridization and immunohistochemistry revealed a widespread distribution, including expression in radial nerve cords, circumoral nerve ring, digestive system, tube feet and innervation of interossicular muscles. In vitro pharmacology revealed that the ArPPLNP2‐derived neuropeptide ArPPLN2h has no effect on the contractility of tube feet or the body wall‐associated apical muscle, contrasting with the relaxing effect of ArPPLN1b (ArSMP) on these preparations. ArPPLN2h does, however, cause dose‐dependent relaxation of cardiac stomach preparations, with greater potency/efficacy than ArPPLN1b and with similar potency/efficacy to the SALMFamide neuropeptide S2. In conclusion, there are similarities in the expression patterns of ArPPLNP1 and ArPPLNP2 but our data also indicate specialization in the roles of neuropeptides derived from these two PP/OK‐type precursors in starfish.

## 1 INTRODUCTION

Molluscan pedal peptides (PPs) and arthropodan orcokinins (OKs) are structurally related neuropeptides that are the prototypes of a family of neuropeptides that have been identified in several phyla, including other protostomes (nematodes, annelids), and deuterostomes (echinoderms). Thus, the evolutionary origin of PP/OK‐type neuropeptides can be traced to the bilaterian common ancestor of protostomes and deuterostomes (Jekely, 2013; Rowe & Elphick, 2012). However, little is known about the physiological roles of PP/OK‐type neuropeptides in non‐molluscan/arthropodan phyla.

PP was discovered in the mollusk *Aplysia californica* on account of its expression by neurons in the pedal ganglia (Lloyd & Connolly, 1989). Subsequently, molecular analysis of *Aplysia* and other molluscan species has revealed the occurrence of multiple precursor proteins that each comprise “cocktails” of structurally related peptides (Moroz et al., 2006; Veenstra, 2010, 2011). Investigation of the effects of PPs in mollusks has revealed that these neuropeptides regulate foot muscle activity and increase the ciliary beat frequency of cells in the foot epithelium, actions that are indicative of physiological roles in the control of locomotor activity (Araki, Liu, Zhang, Takeuchi, & Munekata, 1995; Hall & Lloyd, 1990; Malyshev, Norekian, & Willows, 1999; Willows, Pavlova, & Phillips, 1997).

OK was identified in the crayfish *Orconectes limosus* as a neuropeptide that enhances the frequency and amplitude of spontaneous contractions of the hind‐gut (Stangier, Hilbich, Burdzik, & Keller, 1992). Subsequent studies have revealed that OKs also alter the rhythmic motor output of the stomatogastric nervous system in crustaceans (Li et al., 2002; Skiebe, Dreger, Meseke, Evers, & Hucho, 2002). Furthermore, experimental studies on insects have revealed that OKs cause a phase‐dependent shift in circadian locomotor activity in cockroaches (Hofer & Homberg, 2006), regulate “awakening” behavior in the beetle *Tribolium castaneum* (Jiang, Kim, & Park, 2015) and regulate ecdysis in the kissing bug *Rhodnius prolixus* (Wulff et al., 2017). Thus, PP/OK‐type neuropeptides have a variety of actions in mollusks and arthropods but with stimulatory actions appearing to be a common theme.

Discovery of two genes in the sea urchin *Strongylocentrotus purpuratus* (phylum Echinodermata) that encode precursors of PP‐like neuropeptides (SpPPLNP1, SpPPLNP2) provided the first evidence that PP/OK‐type peptides also occur in deuterostomes (Rowe & Elphick, 2012). However, nothing is known about the physiological roles of PP/OK‐type neuropeptides in sea urchins. Recently, a neuropeptide isolated from the starfish *Patiria pectinifera* that acts as a muscle relaxant (“starfish myorelaxant peptide” or SMP) was identified as a PP/OK‐type neuropeptide (Kim et al., 2016). Cloning and sequencing of a cDNA encoding the SMP precursor revealed that it comprises 12 copies of SMP and 7 copies of three other SMP‐like peptides. Analysis of the distribution of SMP precursor transcripts in *P. pectinifera* using quantitative PCR revealed a widespread pattern of expression. Furthermore, in vitro pharmacological experiments revealed that SMP causes relaxation of three preparations from *P. pectinifera*—the apical muscle, tube feet, and cardiac stomach. Collectively, these data indicated that SMP may have a general role as a muscle relaxant in starfish (Kim et al., 2016).

A homolog of the *P. pectinifera* SMP precursor comprising 8 copies of SMP‐like PP/OK‐type peptides has been identified in the common European starfish *Asterias rubens* (Kim et al., 2016). Furthermore, a partial sequence of a second PP/OK‐type precursor has also been identified in *A. rubens* (Semmens et al., 2016). Therefore, we have named the *A. rubens* SMP‐type precursor *A. rubens* PP‐like neuropeptide precursor 1 (ArPPLNP1) and we have named the other *A. rubens* PP/OK‐type precursor *A. rubens* PP‐like neuropeptide precursor 2 (ArPPLNP2).

Recently, we reported a detailed analysis of the anatomy of ArPPLNP1 expression in *A. rubens* using mRNA in situ hybridization and immunohistochemistry (Lin, Egertová, Zampronio, Jones, & Elphick, 2017). Consistent with qPCR data from *P. pectinifera* (Kim et al., 2016), a widespread pattern of expression of ArPPLNP1 and the neuropeptides derived from this precursor was revealed in *A. rubens*. Thus, cells expressing ArPPLNP1 transcripts were detected, for example, in the radial nerve cords, circumoral nerve ring, esophagus, cardiac stomach, pyloric stomach, pyloric caeca, tube feet, and body wall (Lin, Egertová et al., 2017). Immunohistochemical analysis using antibodies to one of the neuropeptides derived from ArPPLNP1 (ArPPLN1b or ArSMP) revealed a pattern of immunostained cells consistent with data obtained using mRNA in situ hybridization. Furthermore, immunohistochemistry revealed extensive networks of immunostained processes in the radial nerve cords, circumoral nerve ring, digestive system, tube feet, coelomic lining, apical muscle, and body wall (Lin, Egertová, et al., 2017). One of the most striking features of the immunostaining was the presence of labeled processes in close association with interossicular muscles that link the calcite ossicles of the endoskeleton. Consistent with this pattern of expression, intense immunostaining was also observed in the lateral motor nerves, components of the starfish nervous system that were first described based on analysis of histochemical staining (Smith, 1937). Investigation of the in vitro pharmacological effects of ArPPLN1b revealed that it causes dose‐dependent relaxation of three preparations from *A. rubens*—apical muscle, tube feet and cardiac stomach (Lin, Egertová et al., 2017), consistent with previous findings from *P. pectinifera* (Kim et al., 2016). Thus, collectively the data obtained from experimental studies on SMP/PPLN1‐type neuropeptides in *P. pectinifera* and *A. rubens* indicate that a physiological role of these peptides is to act as inhibitory neuromuscular transmitters or modulators in starfish.

The objective of this study was to investigate the expression pattern and pharmacological actions of neuropeptides derived from the second PP/OK‐type neuropeptide precursor in *A. rubens*, ArPPLNP2.

## 2 METHODS

### 2.1 Animals

Starfish (*A. rubens*) with a diameter >4 cm were collected at low tide from the Thanet coast (Kent, UK) or were obtained from a fisherman based at Whitstable (Kent, UK). These animals were maintained in a circulating seawater aquarium at ∼12°C in the School of Biological and Chemical Sciences at Queen Mary University of London and were fed on mussels (*Mytilus edulis*). Smaller juvenile specimens of *A. rubens* (diameter 0.5–1.5 cm) were collected at the University of Gothenberg Sven Lovén Centre for Marine Infrastructure (Kristenberg, Sweden) and fixed in Bouin's solution.

### 2.2 cDNA cloning and sequence analysis

Analysis of *A. rubens* radial nerve cord transcriptome sequence data has enabled identification of a contig encoding the partial sequence (325 residues) of PP‐like neuropeptide precursor 2 (ArPPLNP2), which comprises a 31‐residue N‐terminal signal peptide and seven putative neuropeptides bounded by dibasic cleavage sites (Semmens et al., 2016; GenBank Accession number KT601720). To facilitate cloning and sequencing of the complete open reading frame (ORF) of ArPPLNP2, ovarian transcriptome sequence data obtained from other starfish species were analyzed (Reich, Dunn, Akasaka, & Wessel, 2015; http://www.echinobase.org/Echinobase/Blasts) and a contig (GAUS01023767.1) comprising the 3′ region of PPLNP2 was identified in *Asterias forbesi*. Combining the partial PPLNP2 transcript sequences from *A. rubens* and *A. forbesi*, forward and reverse primers (5′‐GAGACATCGAGGGTGGTTTTG‐3′, 5′‐GGCCCGGCCTAAGAATCAT‐3′) were designed to enable PCR amplification of a full‐length ArPPLNP2 cDNA from *A. rubens*.

The methods used for RNA extraction, cloning and sequencing were as described previously (Lin, Mita et al., 2017), but with minor modifications. Zero Blunt TOPO PCR cloning kit (Invitrogen, Carlsbad, CA) was used to ligate the PCR product into the pCR‐Blunt II with TOPO vector for sequencing of a ArPPLNP2 cDNA comprising the entire ORF. T‐easy vector (Promega) was employed for cloning a cDNA comprising a partial ORF of ArPPLNP2, using the following primers: 5′‐CTATTCTGTCTGGCTCTT‐3′ (forward) and 5′‐GACAGCTTCTTCTCTCTTA‐3′ (reverse).

### 2.3 Mass spectrometric identification of peptides derived from ArPPLNP2 in extracts of *A. rubens* radial nerves

The structures of neuropeptides derived from ArPPLNP2 were determined by MS/MS analysis of *A. rubens* radial nerve cords extracted in 90% methanol/9% acetic acid, employing use of methods described previously (Lin, Egertová et al., 2017; Lin, Mita et al., 2017).

### 2.4 Localization of ArPPLNP2 expression in *A. rubens* using mRNA in situ hybridization

A cDNA comprising a partial ORF of ArPPLNP2 was used as a template for synthesis of probes for mRNA in situ hybridization. The methods employed for (a) production of anti‐sense and sense DIG‐labeled RNA probes, (b) preparation of sections of fixed specimens (diameter 4–6 cm) of *A. rubens* and (c) visualization of ArPPLNP2 transcripts in sections of *A. rubens* were the same as reported previously in a study investigating expression of the relaxin‐like gonad‐stimulating peptide precursor in *A. rubens* (AruRGPP) (Lin, Mita et al., 2017).

### 2.5 Production and characterization of rabbit antibodies to ArPPLNP2‐derived neuropeptides

The peptide ArPPLN2h (GRTSLSGSSGLTHLSSGFH) was selected as a target antigen for production of rabbit antibodies because it is the most abundant of the ArPPLN2‐derived neuropeptides, occurring in triplicate whilst other peptides occur singly in the precursor. Furthermore, ArPPLN2h shares between 58 and 95% sequence identity with the other ArPPLNP2‐derived neuropeptides. A peptide comprising the C‐terminal octapeptide of ArPPLN2h but with the addition of N‐terminal lysine and tyrosine residues (KYTHLSSGFH) was designed as an antigen (ag) peptide (ArPPLN2h‐ag) and synthesized by Peptide Protein Research Ltd. (Hampshire, UK). Rabbit immunization with the ArPPLN2h‐ag–thyroglobulin conjugate and serum collection was performed by Charles River Labs (Margate, UK), as described previously for ArPPLN1b (Lin, Egertová et al., 2017).

To assess production of antibodies during the immunization protocol and following collection of a terminal bleed, antisera were tested for antibodies to ArPPLN2h‐ag using Enzyme‐Linked ImmunoSorbent Assays (ELISA), employing the same methods as described previously for ArPPLN1b (Lin, Egertová et al., 2017).

### 2.6 Localization of ArPPLN2h in *A. rubens* using immunohistochemistry

The methods employed for (a) preparation of sections of fixed specimens (diameter 4–6 cm) of *A. rubens*, (b) visualization of ArPPLN2h expression in sections of *A. rubens* using immunohistochemistry (including pre‐absorption tests) and (c) photography of immunostained sections and preparation of montages were the same as those reported previously for ArPPLN1b (Lin, Egertová et al., 2017).

### 2.7 In vitro and in vivo pharmacology

Consistent with the selection of ArPPLN2h as a representative neuropeptide for generation of antibodies (see above), ArPPLN2h was also selected as a representative ArPPLNP2‐derived peptide for in vitro and in vivo pharmacological experiments. ArPPLN2h was synthesized by Peptide Protein Research Ltd. (Hampshire, UK) and was tested on in vitro preparations of the cardiac stomach, apical muscle and tube foot from *A. rubens*, using the same methods reported previously for ArPPLNP1b (Lin, Egertová et al., 2017). The SALMFamide neuropeptide S2 (SGPYSFNSGLTF‐NH_2_), which is a known relaxant of the cardiac stomach, apical muscle, and tube feet in *A. rubens* (Melarange & Elphick, 2003; Melarange, Potton, Thorndyke, & Elphick, 1999), was tested in parallel with ArPPLN2h both as a positive control and to enable comparative analysis of potency/efficacy. A previous study demonstrated that S2 causes eversion of the cardiac stomach in *A. rubens* when injected in vivo (Melarange et al., 1999). Therefore, here the effect of in vivo injection of ArPPLN2h in *A. rubens* was investigated by intracoelomic injection of starfish (*n* = 10) with 100 µL of 1 mM ArPPLN2h.

## 3 RESULTS

### 3.1 Cloning and sequencing of a cDNA encoding ArPPLNP2

**Figure 1:**
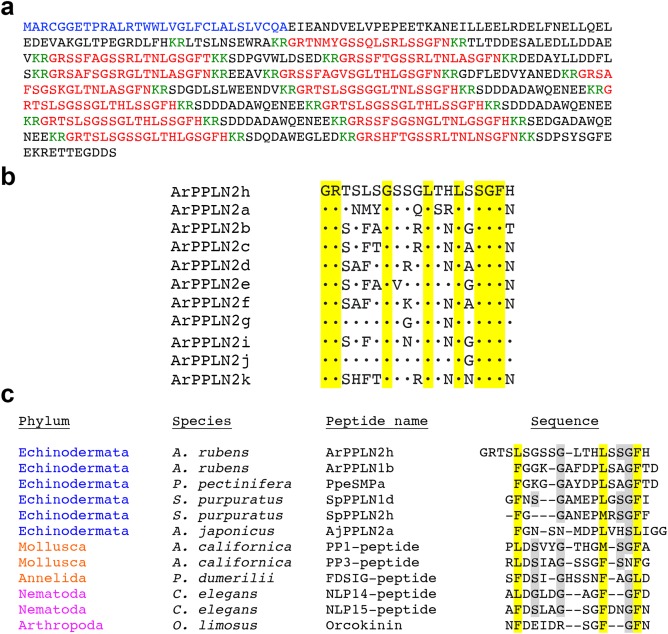
Sequence of ArPPLNP2 and alignments of predicted ArPPLNP2‐derived peptides. (a) Sequence of ArPPLNP2 with the predicted signal peptide marked in blue, predicted cleavage sites marked in green, and peptides ArPPLN2 a to k marked in red. (b) Alignment of ArPPLN2h, which occurs in triplicate in ArPPLNP2, with other peptides derived from ArPPLNP2, which occur singly in ArPPLNP2, as shown in (a). All of the peptides comprise nineteen residues, and residues that are identical to the corresponding residue in ArPPLN2h are shown with a black dot. Eight of the residues in ArPPLN2h are conserved in the ten other peptides (highlighted in yellow). (c) Manual alignment of ArPPLN2h, as a representative ArPPLNP2‐derived peptide, with ArPPLN1b and with PP/OK‐type peptides from other species. Hydrophobic residues (Phe, Leu or Met) are conserved at three positions (highlighted in yellow), one located in the N‐terminal region and two located in the C‐terminal region. Other residues that are conserved between peptides from at least one deuterostome (echinoderm) species and at least one protostome species are highlighted in gray. Citations and/or accession numbers for the peptide sequences included here are as follows: ArPPLN2h (this article; KT601720); ArPPLN1b (Kim et al., 2016; Lin, Egertová et al., 2017; KT870153); PpeSMPa (Kim et al., 2016; KT870152); SpPPLN1d (Rowe & Elphick, 2012; XP_785647); SpPPLN2h (Rowe & Elphick, 2012; XP_003727926); AjPPLN2a (Rowe et al., 2014; Isotig 17873); PP1‐peptide (Lloyd & Connolly, 1989; Moroz et al., 2006; NP_001191585); PP3‐peptide (Moroz et al., 2006; NP_001191625); NLP14‐peptide (Nathoo, Moeller, Westlund, & Hart, 2001; NP_001257067); NLP15 peptide (Nathoo et al., 2001; T20275); OK (Stangier et al., 1992; P37086)

A cDNA encoding ArPPLNP2 was cloned and sequenced (GenBank accession number KT601720). The ArPPLNP2 ORF encodes a 563 amino acid residue protein comprising a 31‐residue signal peptide and 13 copies of predicted PP/OK‐type peptides, which we have named ArPPLN2a‐k according to their position in the precursor protein. There are three copies of ArPPLN2h and one copy of each of the other peptides (Figure 1a,b). As illustrated in Figure 1b, all the peptides have the conserved motifs GR(T/S) and LT(H/N)LXSGF(H/N) (where X is variable) located N‐terminally and C‐terminally, respectively. Furthermore, comparison of ArPPLN2h, as a representative peptide, with other PP/OK‐type peptides revealed sequence conservation at several positions, but most notably three hydrophobic residues: Leu^5^ located in the N‐terminal region and Leu^14^ and Phe^18^ located in the C‐terminal region (Figure 1c).

### 3.2 Mass spectrometric identification of ArPPLNP2‐derived peptides in *A. rubens* radial nerve cords extracts

**Figure 2:**
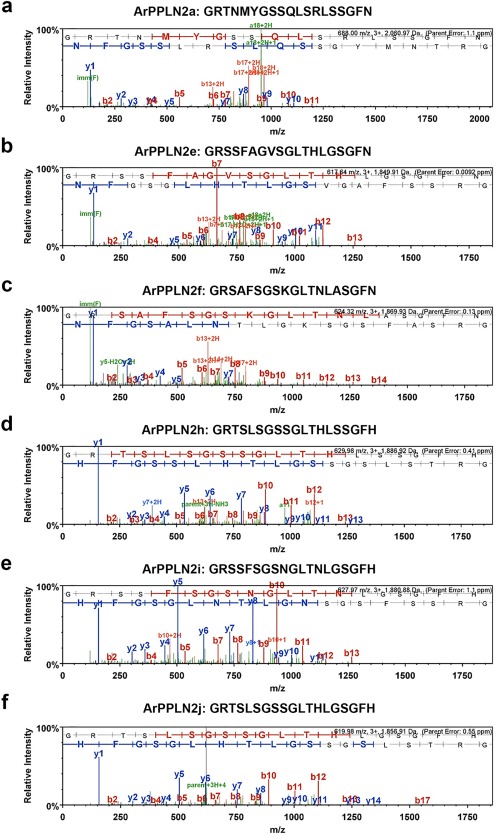
Mass spectrometric identification of ArPPLN2 peptides in an extract of *A. rubens* radial nerve cords. (a–f) Annotated MS/MS spectra for ArPPLN2a, e, f, h, i, j, respectively. The b series of peptide fragment ions are shown in red, the y series in blue and additional identified peptide fragment ions in green. The amino acid sequence identified in the mass spectrum is highlighted at the top of the figures. MS/MS spectra are shown for (a) ArPPLN2a (Mascot score 31.30), (b) ArPPLN2e (Mascot score 38.01), (c) ArPPLN2f, (Mascot score 64.86), (d) ArPPLN2h, (Mascot score 67.8), (e) ArPPLN2i, (Mascot score 65.62) and (f) ArPPLN2j, (Mascot score 67.47)

Ten of the eleven predicted peptides derived from ArPPLNP2 (Figure 1a,b) were detected by MS/MS in an acidified methanol extract of radial nerve cord without additional protease (trypsin) treatment. All precursor ions were detected with less than 2 ppm mass deviation from predicted sequences. ArPPLN2a, e, f, h, i, j were detected with high quality MS/MS data (Figure 2). ArPPLN2b, d, k were detected with poor fragmentation in the MS/MS data, only partial coverage of ArPPLN2g was observed and ArPPLN2c was not detected at all (data not shown).

### 3.3 Localization of ArPPLNP2 transcripts in *A. rubens* using mRNA in situ hybridization

**Figure 3:**
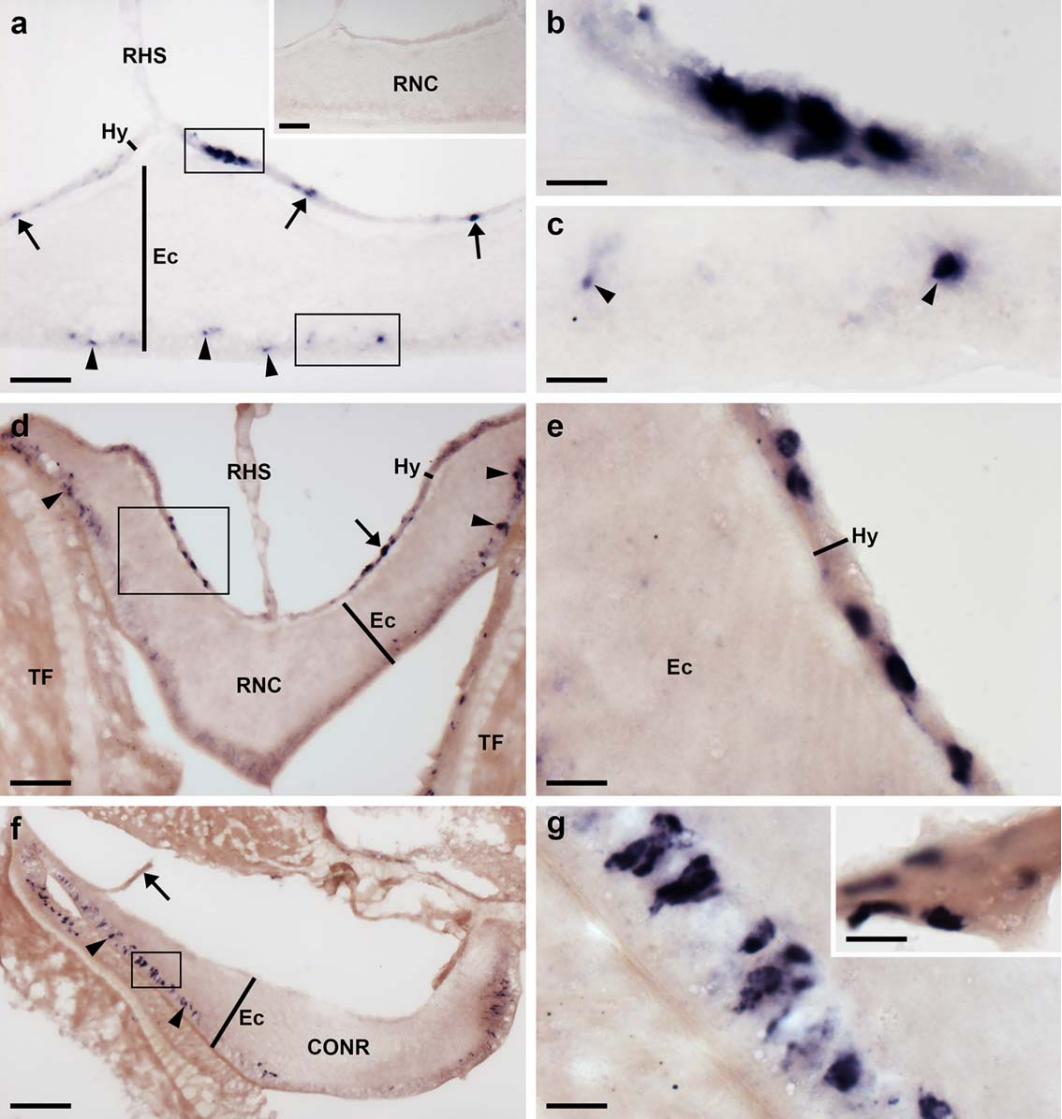
Localization of ArPPLNP2 mRNA in the radial nerve cord and circumoral nerve ring of *A. rubens* using in situ hybridization. (a) Longitudinal parasagittal section of the radial nerve cords incubated with anti‐sense probes showing groups of stained cells interspersed along the length of the nerve cord in the hyponeural (rectangle and arrows) and ectoneural regions (rectangle and arrowheads). Higher magnification images of the boxed regions are shown in (b) and (c). The inset of panel (a) shows the absence of staining in a longitudinal parasagittal section of a radial nerve cord incubated with sense probes, demonstrating the specificity of staining observed with anti‐sense probes. (d) Transverse section of radial nerve cord showing groups of stained cells in the hyponeural (rectangle and arrow) and ectoneural regions (arrowheads); a higher magnification image of the boxed region is shown in (e). (f) Transverse section of circumoral nerve ring showing stained cells in the epithelium of the ectoneural region (arrowheads). A higher magnification image of the boxed region is shown in (g). Note that in (f) no hyponeural staining is evident because the hyponeural tissue is damaged (arrow). A high magnification image of stained cells in a detached hyponeural region of the circumoral nerve ring is shown in the inset of panel (g). CONR, circumoral nerve ring; Ec, ectoneural region of radial nerve cord; Hy, hyponeural region of radial nerve cord; RHS, radial hemal strand; RNC, radial nerve cord; TF, tube foot. Scale bars: 50 μm in (a), (a) inset, (d), (f); 10 μm in (b), (c), (e), (g), (g) inset

**Figure 4:**
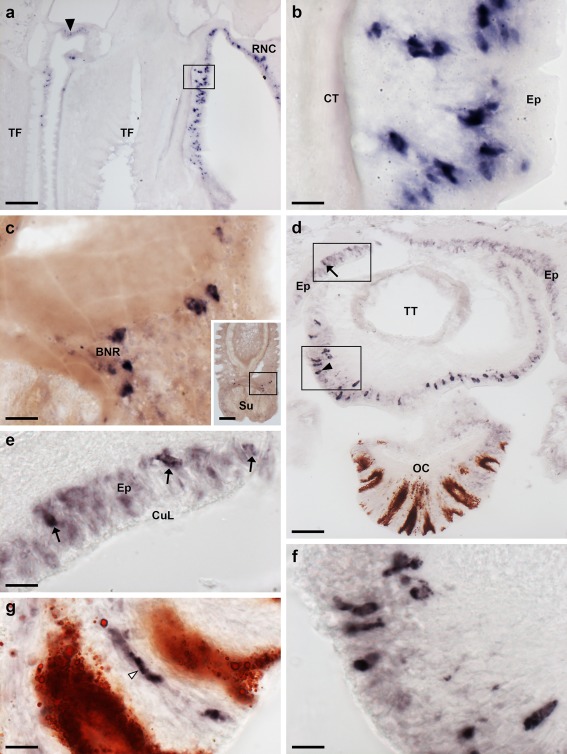
Localization of ArPPLNP2 mRNA in tube feet and the arm tip region of *A. rubens* using in situ hybridization. (a) Longitudinal section of a tube foot showing stained cells at the junction with an adjacent tube foot (arrow) and in the sub‐epithelial layer near to the junction with the adjacent radial nerve cord (rectangle), which is shown at higher magnification in (b). (c) Stained cells located near to the tube foot basal nerve ring. The inset shows the location of the stained cells (rectangle) in a lower magnification image of the tube foot. (d) Transverse cryostat section of an arm tip showing the pigmented optic cushion and terminal tentacle. Stained cells can be seen in the terminal tentacle external epithelium (rectangle with arrowhead) and in the body wall epithelium (rectangle with arrow). The boxed areas in (d) are shown at higher magnification in (e) and (f). (g) High magnification image of the photoreceptor cell layer of an optic cushion showing a stained cell (white arrowhead) between pigmented cells. BNR, basal nerve ring; CT, collagenous tissue; CuL, cuticle layer; Ep, epithelium; OC, optic cushion; RNC, radial nerve cord; Su, sucker; TF, tube foot; TT, terminal tentacle. Scale bars: 100 μm in (a), (c) inset; 10 μm in (b), (c), (e), (f), (g); 50 μm in (d)

**Figure 5:**
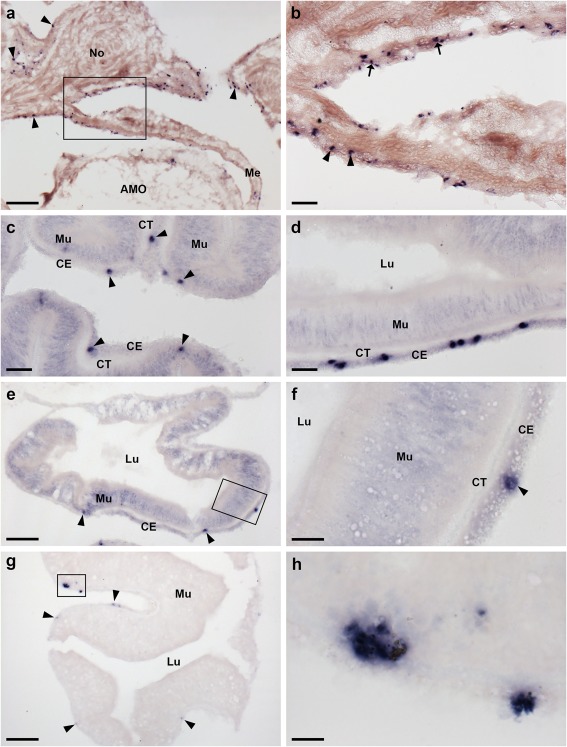
Localization of ArPPLNP2 mRNA in the digestive system of *A. rubens* using in situ hybridization. (a) Transverse section of an arm showing stained cells (rectangle and arrowheads) in the coelomic epithelium of a cardiac stomach extrinsic retractor strand nodule and in mesenteries that attach the extrinsic retractor strands to the ambulacral ossicles. The boxed region is shown at higher magnification in (b), where stained cells can be seen in the coelomic epithelium of a mesentery (arrowheads) and in the coelomic epithelium of a nodule. (c) Section of a cardiac stomach showing stained cells (arrowheads) in the coelomic epithelium. (d) Section of pyloric stomach showing stained cells in the coelomic epithelium. (e, f) Transverse section of a pyloric duct showing stained cells (rectangle and arrowheads) in the coelomic epithelium. The boxed region in (e) is shown at higher magnification in panel (f). (g, h) Transverse section of a pyloric caecum diverticulum showing stained cells (rectangle and arrowheads) in the coelomic epithelium layer. The boxed region in (g) is shown at higher magnification in panel (h). AMO, ambulacral ossicle; CE, coelomic epithelium; CT, collagenous tissue; Lu, lumen; Me, mesentery; Mu, mucosa; No, nodule. Scale bars: 100 μm in (a), (g); 20 μm in (b), (c), (d); 50 μm in (e); 10 μm in (f), (h)

Analysis of the distribution of ArPPLNP2 transcripts in *A. rubens* revealed a widespread pattern of expression, including stained cells in the radial nerve cords and circumoral nerve ring (Figure 3), tube feet and terminal tentacle (Figure 4) and digestive system (Figure 5). Readers unfamiliar with starfish anatomy are referred to an introductory overview of the anatomy of *A. rubens* reported previously (Lin, Egertová et al., 2017).

In longitudinal sections of the radial nerve cords, stained cells can be seen as clusters in the hyponeural region (Figure 3a,b), whilst in the ectoneural region stained cells were observed quite sparsely along the length of the epithelial layer (Figure 3a,c). The specificity of the staining observed with anti‐sense probes (Figure 3a) was demonstrated by control experiments where no staining was observed with sense probes (Figure 3a, inset). Likewise, the specificity of staining observed with anti‐sense probes in other regions of the body (see below) was confirmed by tests with sense probes, where no staining was observed (data not shown). Transverse sections of the radial nerve cords revealed stained cells in the hyponeural region and in the lateral ectoneural region (Figure 3d,e). Transverse sections of the circumoral nerve ring revealed that stained cells are largely concentrated in the outer and inner regions of the ectoneural epithelium, proximal to peri‐oral tube feet and the peristomial membrane, respectively (Figure 3f,g). Stained cells are also present in the hyponeural region of the circumoral nerve ring (Figure 3g, inset), but because this tissue is sometimes damaged or lost during tissue processing the stained hyponeural cells are not always seen (Figure 3f).

In tube feet, stained cells were observed in a sub‐epithelial position proximal to the radial nerve cord (Figures 3d, 4a,b) or circumoral nerve ring (Figure 3f) or between adjacent tube feet (Figure 4a). Stained cells are also present in the tube foot sucker, closely associated with the basal nerve ring (Figure 4c). In the tube foot‐like terminal tentacles stained cells were observed in or below the external epithelial layer (Figure 4d,f) and in the body wall epithelium that surrounds the terminal tentacle (Figure 4d,e). Stained cells were also observed in the photoreceptor cell layer of the optic cushion (Figure 4g), which is located at the base of each terminal tentacle.

The cardiac stomach is attached to the ambulacrum in each of the five arms of *A. rubens* by extrinsic retractor strands. The paired extrinsic retractor strands coalesce to form a nodule that connects the extrinsic retractor strands with the intrinsic retractor strands located within the wall of the cardiac stomach (Anderson, 1954). Large numbers of ArPPLNP2‐expressing cells were observed in the coelomic lining of the nodule and in the ceolomic lining of extrinsic retractor strands and mesenteries that are attached to the ambulacral ossicles (Figure 5a,b). ArPPLNP2‐expressing cells were also revealed in the coelomic lining of the cardiac stomach (Figure 5c), pyloric stomach (Figure 5d), pyloric duct (Figure 5e,f) and pyloric caeca (Figure 5g,h).

### 3.4 Characterization of a rabbit antiserum to ArPPLN2h using ELISA

ELISA analysis revealed that ArPPLN2h‐ag immunoreactivity was detected with antiserum at dilutions ranging from 10^−3^ to 10^−6^ and no cross‐reactivity with ArPPLN1b. Furthermore, with an antiserum dilution of 10^−4^ the ArPPLN2h‐ag peptide was detected in the range from 10^−10^ to 10^−12^ moles, indicative of a high titer of specific antibodies to the ArPPLN2h‐ag peptide. The rabbit antiserum to ArPPLN2h has been assigned the RRID AB_2716765.

### 3.5 Immunohistochemical localization of neuropeptides derived from ArPPLN2 in *A. rubens*

#### 3.5.1 Radial nerve cords, circumoral nerve ring, marginal nerve cords and lateral motor nerves

**Figure 6:**
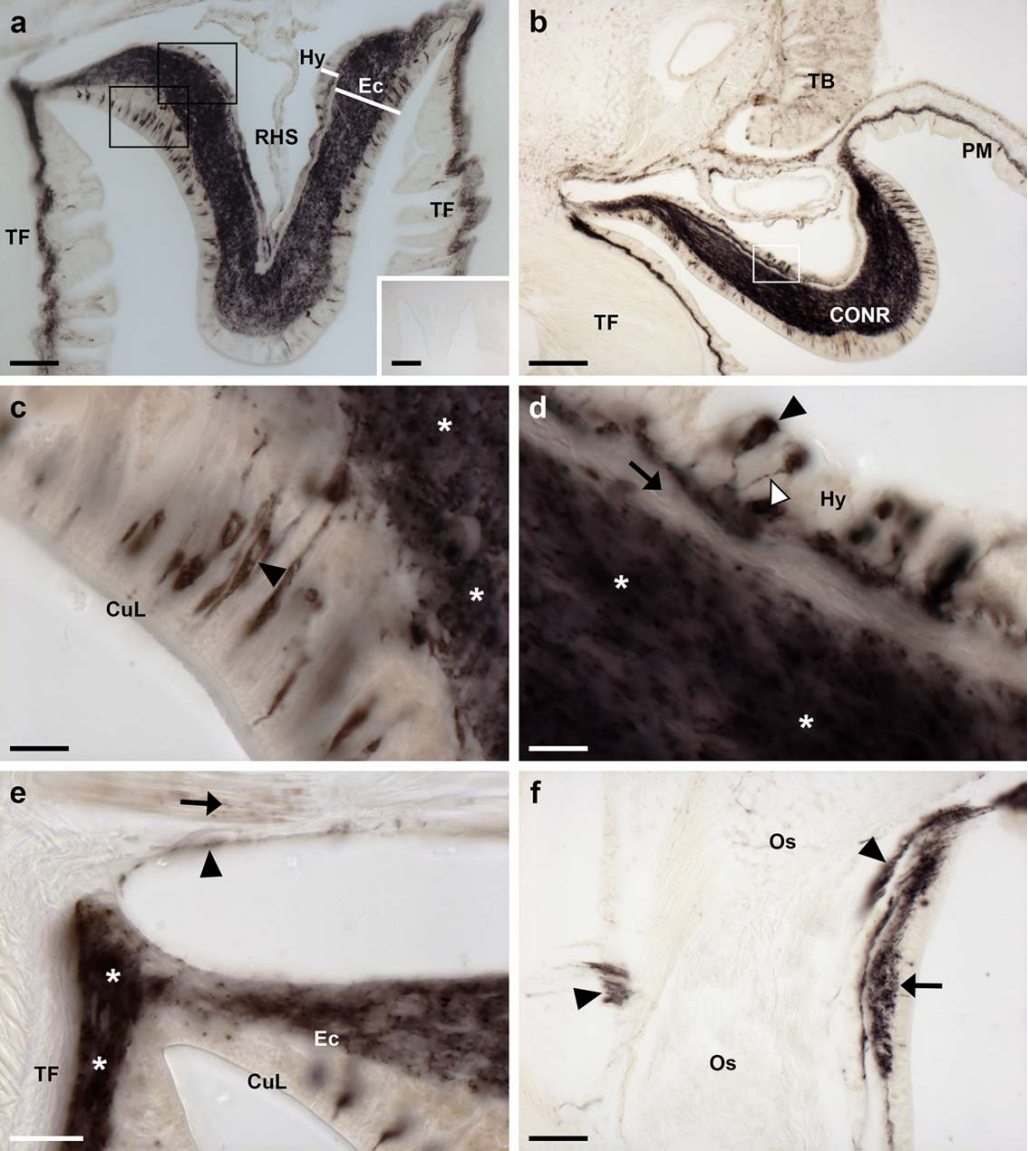
Localization of ArPPLN2h‐immunoreactivity (ArPPLN2h‐ir) in the radial nerve cord, the circumoral nerve ring and marginal nerve of *A. rubens*. (a) Transverse section of a radial nerve cord showing ArPPLN2h‐ir in both the ectoneural and hyponeural regions. The inset of (a) shows absence of immunostaining in a radial nerve cord section incubated with ArPPLN1b antiserum pre‐absorbed with the antigen peptide (ArPPLN2h‐ag), demonstrating the specificity of immunostaining observed with the ArPPLN2 antiserum. (b) Immunostaining in a transverse section of the circumoral nerve ring; here immunostained processes can also be seen here in the peristomial membrane, an adjacent oral tube foot and in a Tiedemann's body. A high magnification image of the boxed region can be seen in (d). (c) High magnification image of the ectoneural region of the radial nerve cord showing immunostained bipolar cells in the sub‐cuticular epithelium (arrowheads) and densely packed immunostained processes (asterisks) in the underlying neuropile region. (d). Immunostained monopolar shaped cells (black arrowheads) in the hyponeural region of the circumoral nerve ring, with stained processes (white arrowhead) projecting into a fiber layer that is adjacent to the unstained collagenous tissue layer (arrow). The intensely stained ectoneural neuropile is labeled here with an asterisk. (e) The continuity of immunostaining in the ectoneural region of the radial nerve and in the basiepithelial nerve plexus of an adjacent tube foot (asterisks) can be seen here. The stained process(es) (arrowhead) of a hyponeural neuron(s) can be seen projecting over the roof of the peri‐hemal canal in close association with the transverse infra‐ambulacral muscle (arrow). (f) Immunostaining in the marginal nerve (arrow) and in axonal processes of the lateral motor nerve (arrowheads). CONR, circumoral nerve ring; CuL, cuticle layer; Ec, ectoneural region; Hy, hyponeural region; PM, peristomial membrane; RHS, radial hemal strand; TB, Tiedemann's body; TF, tube foot. Scale bars: 50 μm in (a), (f); 200 μm in (a) inset; 10 μm in (b), (d); 100 μm in (c); 20 μm in (e)

The ArPPLN2h antiserum revealed extensive immunostaining in the radial nerve cords (Figure 6a) and the specificity of this immunostaining was demonstrated in pre‐absorption experiments (Figure 6a, inset). Likewise, the specificity of staining observed with the antiserum in other regions of the body (see below) was confirmed by tests with antiserum pre‐absorbed with the antigen peptide, where no staining was observed (data not shown). Furthermore, pre‐incubation of the antiserum with ArPPLN1b peptide did not affect the staining observed (data not shown), demonstrating that the ArPPLN2h antiserum is not cross‐reactive with a ArPPLNP1‐derived neuropeptide. The pattern of immunostaining in the circumoral nerve ring (Figure 6b) was consistent with that in the radial nerve cords. Bipolar immunostained cells in the ectoneural region of the radial nerve cord are illustrated in Figure 6c and monopolar shaped immunostained cells in the hyponeural region of the circumoral nerve ring are illustrated in Figure 6d. Nerve processes derived from the hyponeural region of the radial nerve cords can be seen to project around the wall of the peri‐hemal canal to innervate the transverse infra‐ambulacral muscle (Figure 6e), whilst immunostained fibers in the ectoneural region are continuous with the basiepithelial nerve plexus of adjacent tube feet (Figure 6e). Lateral to the outer rows of tube feet in each arm are the marginal nerves, which have immunostained cells in an external epithelium and immunostained processes in an underlying neuropile (Figure 6f). Internal to the marginal nerves, immunostaining was observed in the lateral motor nerves (Figure 6f).

#### 3.5.2 Tube feet and terminal tentacle

**Figure 7:**
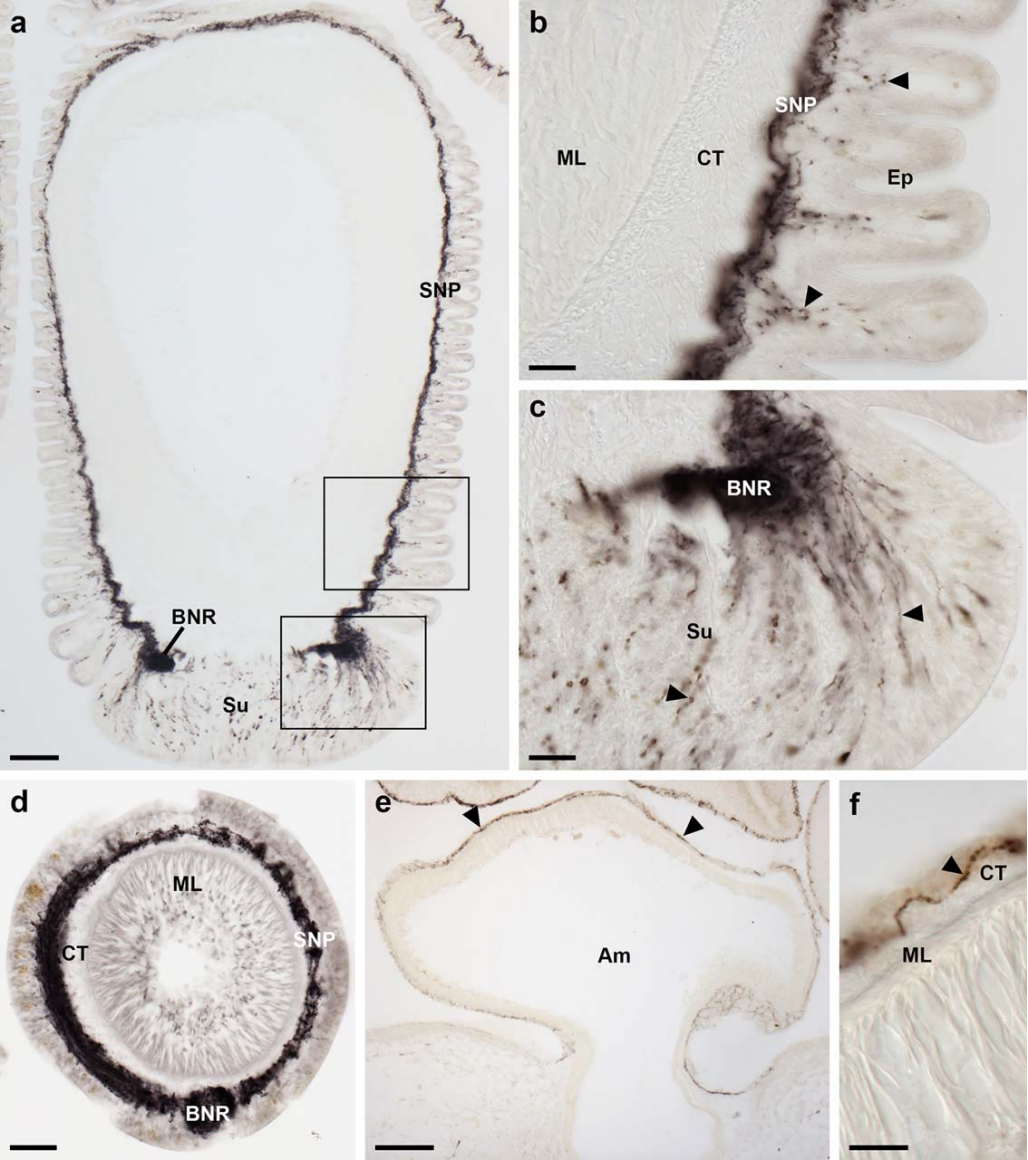
Localization of ArPPLN2h immunoreactivity in tube feet and ampullae of *A. rubens*. (a) Longitudinal section of a tube foot showing immunostaining in the sub‐epithelial nerve plexus, basal nerve ring and sucker. The boxed regions are shown at higher magnification in panels (b, c). (b) Immunostaining in the sub‐epithelial nerve plexus and in processes projecting into epithelial folds of the contracted tube foot (arrowheads). (c) Immunostaining in the basal nerve ring and in processes projecting into the tube foot sucker (arrowheads). (d) Transverse section of a tube foot showing immunostaining in the sub‐epithelial nerve plexus and basal nerve ring. (e, f) Immunostaining in the sub‐epithelial nerve plexus of an ampulla shown at low (e; arrowheads) and high (f; arrowhead) magnification. Am, ampulla; BNR, basal nerve ring; CT, collagenous tissue; Ep, epithelium; ML, muscle layer; SNP, sub‐epithelial nerve plexus; Su, sucker. Scale bars: 100 μm in (a), (e); 20 μm in (b), (c), (d); 10 μm in (f)

**Figure 8:**
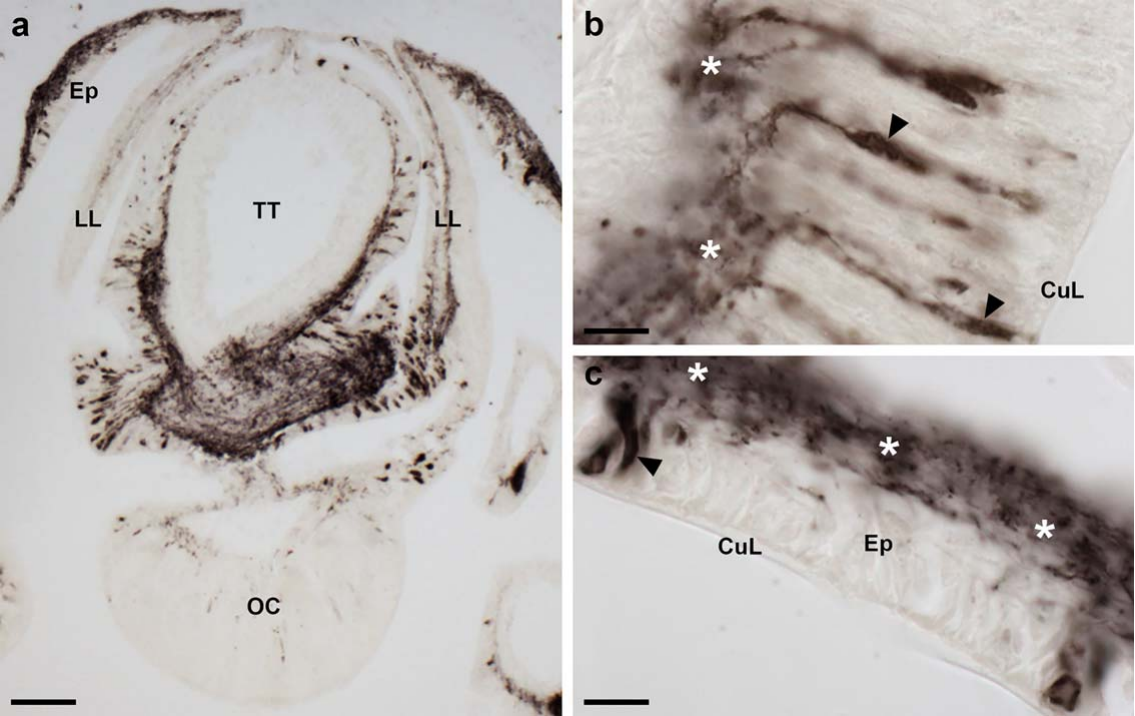
Localization of ArPPLN2h immunoreactivity in the terminal tentacle and associated structures in the arm tip of *A. rubens*. (a) Immunostaining in a transverse section at the base of the terminal tentacle. Stained cells and processes can be seen here in the body wall epithelium, the terminal tentacle and associated lateral lappets and the optic cushion. (b) A high magnification image of arm tip epithelium showing immunostained bipolar cells in the epithelium (arrowhead) and in a dense meshwork of fibers in the underlying basiepithelial plexus (asterisks). (c) High magnification image of the terminal tentacle showing immunostained bipolar cells in the epithelium (arrowheads) with stained processes projecting into a dense meshwork of fibers in the underlying basiepithelial plexus (asterisks). CuL, cuticle layer; Ep, epithelium; LL, lateral lappet; TT, terminal tentacle; OC, optic cushion. Scale bars: 100 μm in (a); 10 μm in (b, c)

Immunostaining is present throughout the sub‐epithelial nerve plexus of tube foot stems (Figure 7a,b) and in the basal nerve ring located in tube foot suckers (Figure 7a,c,d). Immunostained processes can be seen extending from the sub‐epithelial nerve plexus into epidermal folds of the tube foot stem when it is in a contracted state (Figure 7b). Immunostained processes also extend from the basal nerve ring into the tube foot sucker (Figure 7c). Immunostained processes are also present beneath the coelomic epithelium of the tube foot ampulla (Figure 7e,f). In the terminal tentacle, immunostained bipolar cells are present in the external epithelial layer and in the underlying nerve plexus (Figure 8a,b). Stained cells and processes are present in the body wall epithelium and its underlying basiepithelial nerve plexus surrounding the terminal tentacle (Figure 8a, c). Immunostaining is also present in the lateral lappets of the terminal tentacle (Figure 8a) and in the optic cushion, which is located at the base of the terminal tentacle (Figure 8a).

#### 3.5.3 Digestive system

**Figure 9:**
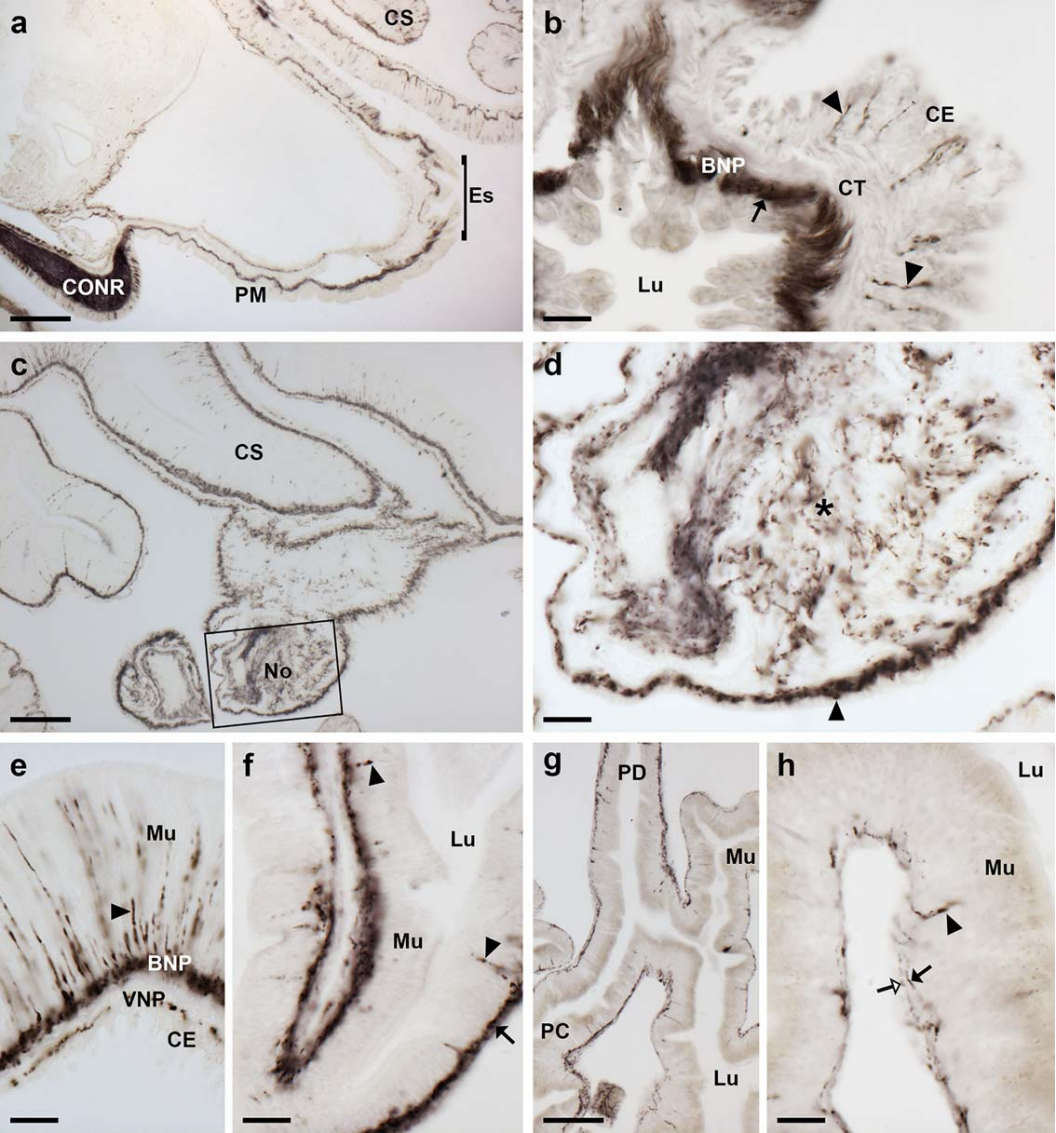
Localization of ArPPLN2h immunoreactivity in the digestive system of *A. rubens*. (a) Transverse section of the central disk showing immunostaining in the peristomial membrane, the esophagus and the cardiac stomach. The intensely stained circumoral nerve ring can also be seen here. (b) High magnification transverse section of the esophagus showing immunostained cells and processes in the coelomic lining (arrowheads) and dense immunostaining in the basiepithelial plexus beneath the lumenal epithelial lining of the esophagus. (c) Immunostaining in the cardiac stomach and in the nodule that links the cardiac stomach to extrinsic retractor strands; a high‐magnification image of the boxed area is shown in (d). (d) Immunostained processes can be seen in the basiepithelial plexus beneath the coelomic lining of the nodule (arrowhead) and in the core of the nodule (asterisk). (e) High magnification image of a cardiac stomach showing immunostained cells in the mucosa (arrowhead) and immunostained processes in the basiepithelial nerve plexus beneath the mucosa and in the visceral nerve plexus beneath the coelomic epithelium. (f) High magnification image of the pyloric stomach showing immunostained cells in the mucosa (arrowheads) and immunostained processes in the basiepithelial nerve plexus (black arrow). (g) Immunostaining in a horizontal section of a pyloric duct and pyloric caecum. (h) High magnification image of a pyloric caecum showing immunostaining in mucosal cell bodies (arrowhead), in the basiepithelial nerve plexus (black arrow) and in the visceral nerve plexus (white arrow). BNP, basiepithelial nerve plexus; CE, coelomic epithelium; CONR, circumoral nerve ring; CS, cardiac stomach; CT, collagenous tissue; Es, esophagus; Lu, lumen; Mu, mucosa; No, nodule; PC, pyloric caeca; PD, pyloric duct; PM, peristomial membrane; VNP, visceral nerve plexus. Scale bars: 200 μm in (a); 20 μm in (b), (d), (e), (f), (h); 100 μm in (c), (g)

The external epithelial layer of the peristomial membrane that surrounds the mouth contains immunostained cells and immunostained processes are present in the underlying basiepithelial nerve plexus (Figure 9a,b). A similar pattern of immunostaining is observed in the short esophagus that is located between the peristomial membrane and the cardiac stomach (Figure 9a).

A widespread pattern of immunostaining was observed in the cardiac stomach (Figure 9c) and in the extrinsic extractor strands that link the cardiac stomach to the ambulacrum in each of the five arms. The paired extrinsic retractor strands coalesce to form a nodule that is located close to a site of insertion on the wall of the cardiac stomach and a dense meshwork of immunolabeled fibers can be seen in the nodule (Figure 9d). Immunostained bipolar cells are present in the mucosa of the cardiac stomach and in the underlying basiepithelial nerve plexus (Figure 9e). Immunostained processes are also present in the visceral nerve plexus located beneath the coelomic epithelium of the cardiac stomach. A similar pattern of immunostaining to that seen in the cardiac stomach is also observed in the pyloric stomach (Figure 9f). The pyloric duct and pyloric ceca also exhibit immunostaining (Figure 9g), with stained processes in the visceral nerve plexus and immunolabeled bipolar cells in the mucosal layer giving rise to immunoreactive fiber networks in the basiepithelial nerve plexus (Figure 9g,h).

#### 3.5.4 Body wall, body wall‐associated appendages and interossicular muscles

**Figure 10:**
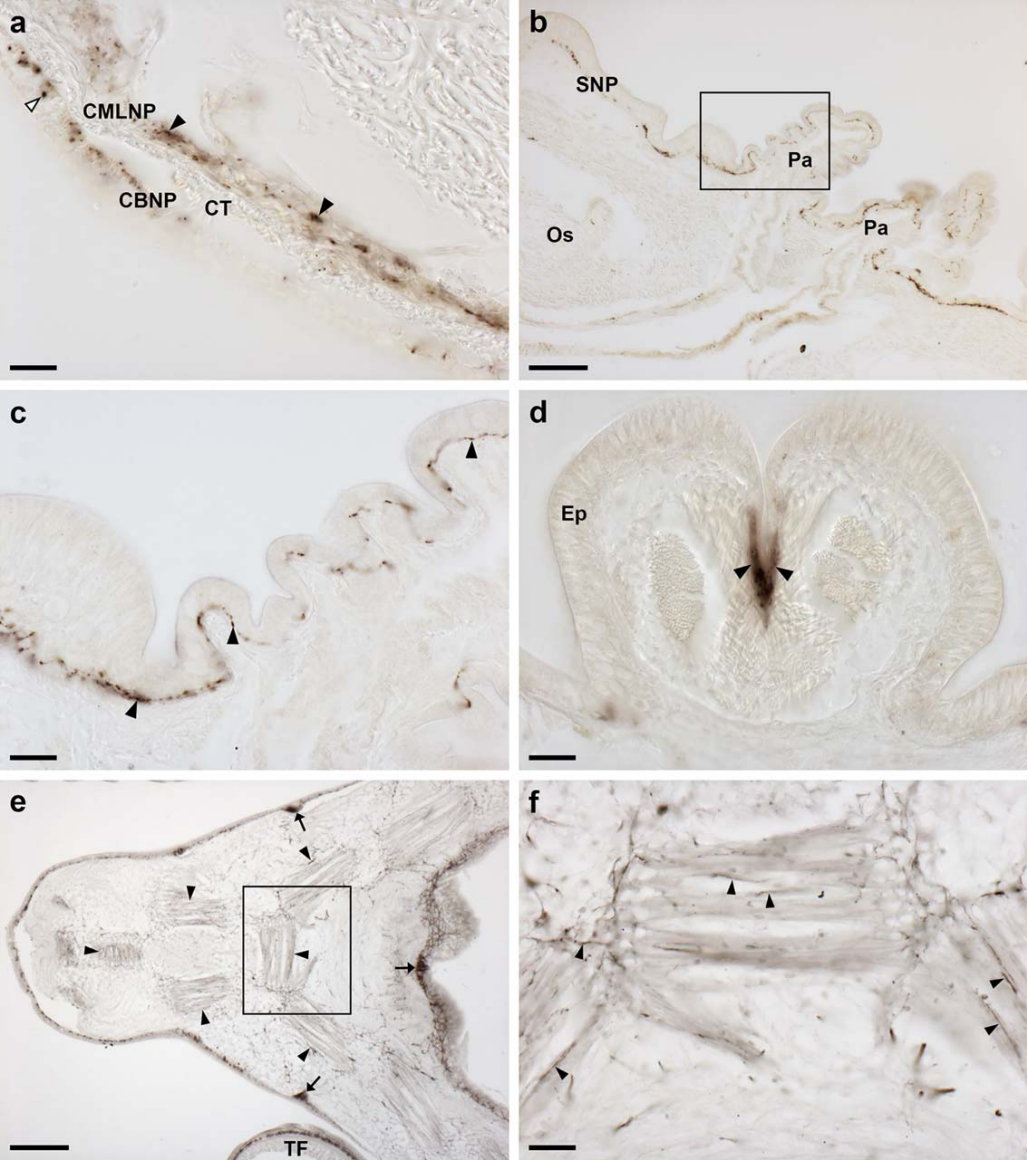
Localization of ArPPLN2h immunoreactivity in the body wall, body wall appendages and interossicular muscles of *A. rubens*. (a) High magnification image of a transverse section of an arm showing the coelomic lining of the body wall, with stained processes in the coelomic basiepithelial nerve plexus (white arrowhead) and in the nerve plexus associated with the circular muscle layer (black arrowheads). (b) Transverse section of an arm showing immunostaining in the sub‐epidermal nerve plexus of the body wall and in papulae. The boxed area is shown at higher magnification in (c), which shows immunostaining in the sub‐epidermal nerve plexus of a papula and adjacent body wall. (d) High magnification image showing immunostained processes associated with adductor muscles (arrowheads) in a pedicellaria. (e, f) Immunostaining in the body wall at the junction between two arms in a horizontal section of a juvenile starfish. Immunostained fibers can be seen associated with muscles that link adambulacral ossicles (arrowheads). Stained fibers are also evident in thickenings of the sub‐epithelial nerve plexus of the body wall (arrows) and in the tube feet. A high magnification image of the boxed region is shown in (f), which shows immunostained processes (arrowheads) in muscles that link adambulacral ossicles. BW, body wall; CBNP, coelomic basiepithelial nerve plexus; CMLNP, circular muscle layer nerve plexus; CT, collagenous tissue; Os, ossicle; Pa, papulae; SNP, sub‐epidermal nerve plexus; TF, tube foot. Scale bars: 20 μm in (a), (c), (d), (f); 100 μm in (b), (e)

ArPPLNP2h‐immunoreactive fibers were observed in the coelomic lining of the body wall (Figure 10a). Thus, stained fibers are present in the coelomic basiepithelial nerve plexus that is located beneath the coelomic epithelium and which is closely associated with a longitudinally orientated muscle layer (Figure 10a,b). Furthermore, immunostained fibers were also observed within the circularly orientated muscle layer of the coelomic lining (Figure 10a,b). Immunostaining of nerve fibers was also observed in the sub‐epidermal plexus of the body wall (Figure 10b,c). This staining extends into papule, hollow finger‐shaped organs located above voids in the calcite skeleton that enable gas exchange between the external seawater and the coelomic fluid. Another type of body wall appendage in starfish is pedicellariae, pincer‐like organs that keep the body surface clear of encrusting material. In pedicellariae, immunostaining was observed in processes associated with the adductor muscles (Figure 10d). Immunostained fibers were also revealed in association with interossicular muscles that link adjacent ossicles of the body wall endoskeleton (Figure 10e,f).

### 3.6 ArPPLN2h has no effect on the contractility of in vitro preparations of apical muscle and tube feet from *A. rubens* but is a potent relaxant of cardiac stomach preparations

**Figure 11:**
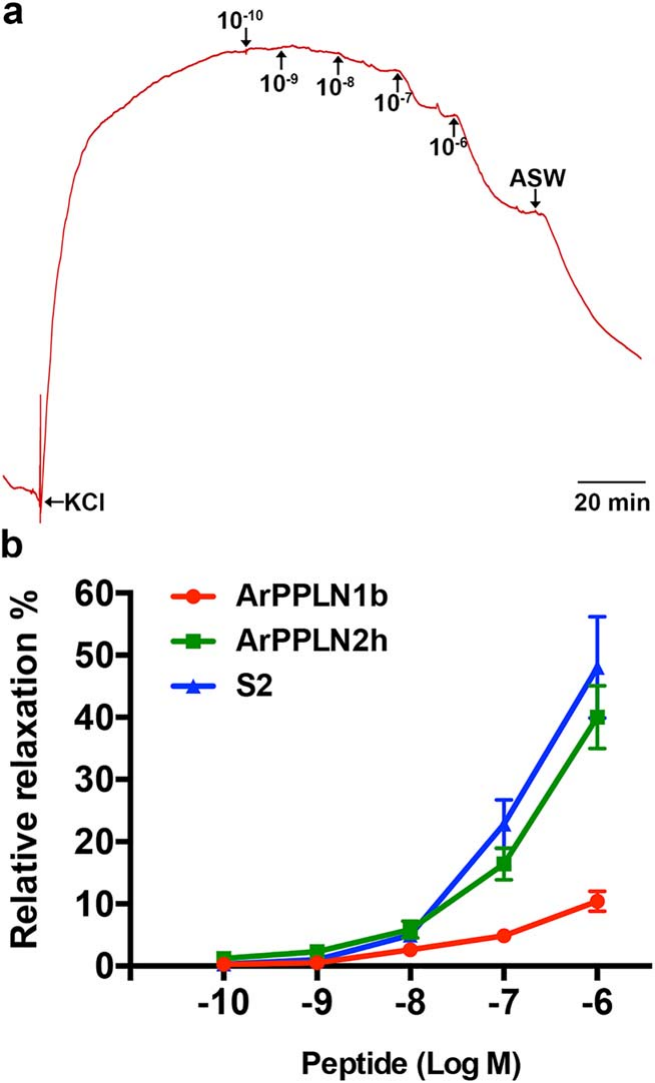
ArPPLN2h causes dose‐dependent relaxation of in vitro preparations of the cardiac stomach from *A. rubens*. (a) Representative recording showing the dose‐dependent relaxing effect of ArPPLN2h (10^−10^ to 10^−6^ M) on a cardiac stomach preparation pre‐contracted with artificial seawater containing 30 mM added KCl (leftward arrow). Following tests with ArPPLN2h, the preparation was washed with artificial seawater (downward arrow). (b) Graph showing concentration‐dependent relaxing effect of ArPPLN2h (green) on cardiac stomach preparations. Each point represents the mean ± *SEM* from at least four separate experiments, with the effect calculated as the percentage reversal of contraction induced by KCl. The effectiveness of ArPPLN2h as a cardiac stomach relaxant is much greater than that of the ArPPLN1b (red) but similar to that of the SALMFamide neuropeptide S2 (blue)

ArPPLN2h was tested at a high concentration (10^−6^ M) on three in vitro preparations from *A. rubens*: apical muscle, tube feet and cardiac stomach. No effects on the contractile state of apical muscle and tube foot preparations were observed, whereas ArPPLN2h caused relaxation of cardiac stomach preparations. ArPPLN2h caused dose‐dependent relaxation of cardiac stomach preparations and comparison of the effects of ArPPLN2h with the SALMFamide neuropeptide S2 revealed that the potency and efficacy of ArPPLN2h was very similar to that of S2 (Figure 11). Thus, relaxing effects were observed with ArPPLN2h and S2 in the concentration range 10^−8^ and 10^−6^ M and the maximum effect (*E*_max_) observed at 10^−6^ M was 40.05 ± 5.07% and 48.03 ± 8.17% reversal of KCl‐induced contraction for ArPPLN2h and S2, respectively.

### 3.7 ArPPLN2h does not induce cardiac stomach eversion

Previous studies revealed that the SALMFamide S2 causes cardiac stomach relaxation in vitro and induces cardiac stomach eversion when injected in vivo (Melarange et al., 1999). Here we observed that ArPPLN2h and S2 cause cardiac stomach relaxation in vitro with similar potency and efficacy. Therefore, we compared the effects of ArPPLN2h and S2 in vivo, with 100 µL of peptide at a concentration of 1 mM injected at two or three sites in the aboral body wall of the arms proximal to the junctions with the central disk region. Cardiac stomach eversion was observed within 5 min of injection in seven out of ten starfish injected with S2 but stomach eversion was not observed in any of ten animals injected with ArPPLN2h.

## 4 DISCUSSION

Here we report the structural, anatomical and functional characterization of neuropeptides derived from *A. rubens* PP‐like neuropeptide precursor 2 (ArPPLNP2). ArPPLNP2 comprises 13 copies of 11 PP/OK‐type peptides (ArPPLN2a‐k), with three copies of ArPPLN2h and one copy each of the other ten peptides. Alignment of ArPPLNP2‐derived peptides with other PP/OK‐type neuropeptides reveals several conserved structural characteristics (see Figure 1c). The functional significance of the conserved residues remains to be determined for starfish PP/OK‐type peptides. However, it is noteworthy that structure‐activity analysis has revealed that N‐ and C‐terminally located phenylalanine residues appear to be critically important for the bioactivity of OK (Bungart, Kegel, Burdzik, & Keller, 1995), consistent with the evolutionary conservation of hydrophobic residues at these positions in PP/OK‐type peptides from other protostomian invertebrates and from echinoderms.

### 4.1 Functional interpretation of the expression patterns and actions ArPPLN2‐type peptides in *A. rubens*

ArPPLNP2 mRNA expressing cells and ArPPLN2h‐immunoreactive cells and processes were detected in both the ectoneural and hyponeural regions of the radial nerve cords and circumoral nerve ring and in the marginal nerves of *A. rubens*. Collectively, these findings indicate that neuropeptides derived from ArPPLNP2 have a widespread role in mediating neuronal signaling in the starfish nervous system. Little is known about the functional organization of the ectoneural nervous system in starfish (or other echinoderms), although it is thought to comprise sensory neurons, interneurons and motor neurons (Cobb, 1970; Cobb, 1987; Mashanov, Zueva, Rubilar, Epherra, & Garcia‐Arraras, 2016). On the other hand, the hyponeural division of the nervous system is recognized as having a purely motor function, based on both anatomical and functional studies (Cobb, 1970; Cobb, 1987; Mashanov et al., 2016). Therefore, the presence ArPPLNP2‐derived neuropeptides in hyponeural cell bodies and processes can be interpreted as evidence of a role in motoneuronal function in starfish. Consistent with this notion, ArPPLN2h‐immunoreactive processes are present in the lateral motor nerves, which innervate interossicular muscles in the starfish body wall (Smith, 1937, 1950). Accordingly, the widespread occurrence of ArPPLN2h‐immunoreactive processes in interossicular muscles and other muscles in *A. rubens* is suggestive of a general role for ArPPLNP2‐derived neuropeptides in neuromuscular signaling.

The presence of ArPPLNP2‐expressing cells and ArPPLN2h‐immunoreactive processes in the basiepithelial nerve plexus of tube feet in *A. rubens* suggested a potential role for ArPPLN2‐derived neuropeptides in the regulation of the contractility of these motile and muscular organs and therefore ArPPLN2h was tested for myoactivity on in vitro preparations of tube feet. ArPPLN2h had no effect on tube foot preparations, contrasting with the ArPPLNP1‐derived neuropeptide ArPPLN1b, which has a pattern of expression in tube feet that is very similar to that of ArPPLN2h but which causes dose‐dependent relaxation of tube foot preparations (Lin, Egertová et al., 2017). These findings are indicative of differences in the physiological roles of ArPPLNP1‐derived and ArPPLNP2‐derived neuropeptides in *A. rubens*. Both ArPPLN1b and ArPPLN2h are also present in the tube foot basal nerve ring and in processes that innervate the adhesive tube foot sucker, which could be indicative of roles in regulation of the secretion of proteins that mediate tube adhesion or detachment (Hennebert, Jangoux, & Flammang, 2013; Hennebert, Wattiez, Waite, & Flammang, 2012; Santos, Haesaerts, Jangoux, & Flammang, 2005).

Each tube foot stem is linked via a tubular canal that extends through the body wall to a bulb‐shaped muscular ampulla, which is located in the perivisceral coelom. Contraction of the ampulla facilitates tube foot extension, whereas relaxation of the ampulla enables accommodation of fluid from the lumen of the podium when a tube foot contracts (Hennebert et al., 2013; Hennebert et al., 2012; Santos et al., 2005). Both ArPPLN1b‐immunoreactive (Lin, Egertová et al., 2017) and ArPPLN2h‐immunoreactive (this study) fibers are present in ampullae and are located in close proximity to the muscle layer. Previous studies have reported that the ampullae are innervated by hyponeural neurons (Smith, 1950) and therefore it is likely that ArPPLN1b‐ and ArPPLN2h‐immunoreactive fibers in the ampulla originate from ArPPLNP1‐ and ArPPLNP2‐expressing hyponeural motoneurons.

The terminal tentacle is a tube foot‐like sensory organ located at the tip of each arm in *A. rubens* and in other starfish. ArPPLN2h‐immunoreactive cells and fibers are present in the wall of the terminal tentacle and in the body wall epithelium and lateral lappets that surround the terminal tentacle. The physiological roles of the lateral lappets (Smith, 1937) are not known, although a possible function would be to act as chemosensory organs. Turning to a different sensory modality, located at the base of the terminal tentacle is a pigmented photosensory organ known as the optic cushion and here ArPPLNP2‐expressing cells are present in the photoreceptor cell layer. It is noteworthy that OKs have a physiological role in mediating light entrainment of circadian activity in insects (Hofer & Homberg, 2006) and therefore it is possible that PP/OK‐type peptides have a similar role in starfish.

Cells expressing ArPPLNP2 transcripts were detected in the coelomic epithelial layer in many regions of the digestive system. In accordance with this pattern of precursor expression, antibodies to ArPPLN2h labeled processes located in the visceral nerve plexus underlying the coelomic epithelium. Extensive immunoreactive fibers in the extrinsic retractor strand nodule of the cardiac stomach were particularly striking, consistent with the abundance of ArPPLNP2‐expressing coelomic epithelial cells lining the nodule. Consistent with the extensive expression of ArPPLN2‐type neuropeptides in the cardiac stomach and other regions of the digestive system, we found that ArPPLN2h caused dose‐dependent relaxation of in vitro cardiac stomach preparations. We previously reported the expression of ArPPLNP1 and neuropeptides derived from this precursor in the cardiac stomach of *A. rubens* and found that ArPPLN1b, like ArPPLN2h, causes relaxation of in vitro preparations of the cardiac stomach (Lin, Egertová et al., 2017). Here we compared the bioactivity of ArPPLN1b and ArPPLN2h as cardiac stomach relaxants and found that ArPPLN2h is much more effective than ArPPLN1b. This difference in the bioactivity of the two peptides may be associated with differences in the patterns of expression of ArPPLNP1‐derived and ArPPLNP2‐derived peptides in the cardiac stomach. Both types of peptides are expressed by mucosal cells and their processes in the basiepithelial plexus, but only ArPPLNP2‐derived peptide (ArPPLN2h) expression was detected in the visceral nerve plexus that underlies the coelomic epithelium of the cardiac stomach. This is significant because the visceral nerve plexus is closely associated with the visceral muscle layer of the cardiac stomach, whereas the basiepithelial nerve plexus is separated from the visceral muscle layer by a layer of collagenous tissue.

Previous studies have revealed that the SALMFamide neuropeptides S1 and S2 cause relaxation of in vitro cardiac stomach preparations from *A. rubens*, but with S2 ten times more potent than S1 (Melarange et al., 1999). Therefore, here we compared the bioactivity of ArPPLN2h with S2 and found that the potency/efficacy of ArPPLN2h is very similar to that of S2 in vitro. In vivo pharmacological tests with S2 have revealed that injection of this peptide into the perivisceral coelom of *A. rubens* triggers eversion of the cardiac stomach, indicating that S2 may be involved in physiological mechanisms that mediate control of stomach eversion when starfish feed extraorally on prey (e.g., mussels) (Melarange et al., 1999), whereas here we found that ArPPLN2h does not trigger cardiac stomach eversion in *A. rubens*. Thus, whilst S2 and ArPPLN2h exhibit similar cardiac stomach relaxing activity in vitro, this appears not to be complemented by the same bioactivity in vivo. This difference in in vivo bioactivity may reflect differences in regions of the cardiac stomach musculature that are regulated by S2 and ArPPLN2h as muscle relaxants and/or differences in the stability of these peptides in vivo. With regard to the latter, it is noteworthy that S2 is a C‐terminally amidated peptide, whereas PP/OK‐type peptides in starfish are not amidated.

### 4.2 Comparison of the expression and bioactivity of ArPPLN1‐type and ArPPLN2‐type peptides in *A. rubens*

Comparison of the expression patterns of ArPPLN1‐type (Lin, Egertová et al., 2017) and ArPPLN2‐type precursors/neuropeptides reveals some general similarities, which may reflect a common evolutionary origin. However, there are also distinct differences in the expression patterns and bioactivity of ArPPLN1‐type and ArPPLN2‐type precursors/neuropeptides. Some of these differences have already been discussed above and here we highlight others. The prototype of PPLN1‐type peptides in starfish is SMP, which was discovered on account of its relaxing effect on the apical muscle of *P. pectinifera* (Kim et al., 2016) and accordingly ArPPLN1b causes dose‐dependent relaxation of apical muscle preparations from *A. rubens* (Lin, Egertová et al., 2017). Furthermore, consistent with the bioactivity of ArPPLN1b, antibodies to ArPPLN1b reveal that this peptide is present in axonal processes that ramify amongst the muscle fibers of the apical muscle (Lin, Egertová et al., 2017). It is interesting, therefore, that in this study no ArPPLN2h‐immunoreactivity was detected in the apical muscle of *A. rubens* and in accordance with this finding ArPPLN2h does not affect the contractile state of the apical muscle in vitro. Thus, whilst both PPLN1‐type and PPLN2‐type peptides act as muscle relaxants in starfish, it appears that they have specialized with respect to the muscle systems that they regulate. Thus, PPLN1‐type peptides act as relaxants of the apical muscle and tube feet, whereas PPLN2‐type peptides do not cause relaxation of these muscle preparations in vitro. Conversely, PPLN2‐type peptides cause relaxation of cardiac stomach preparations with an efficacy and potency comparable to that of the SALMFamide neuropeptide S2, whilst PPLN1‐type peptides are much less efficacious as cardiac stomach relaxants.

### 4.3 Evolution and comparative physiology of PP/OK‐type neuropeptide signaling in the Bilateria

This study of PPLN2‐type peptides in *A. rubens* together with our previous report on PPLN1‐type peptides in *A. rubens* (Lin, Egertová et al., 2017) are the first detailed analyzes of the anatomical expression patterns of PP/OK‐type neuropeptides in an echinoderm. In the future, it would be interesting to perform similar investigations in other starfish. Analysis of the genome/transcriptome of the crown‐of‐thorns starfish *Acanthaster planci* has revealed the presence of genes/transcripts encoding proteins that are orthologs of ArPPLNP1 and ArPPLNP2 (Smith et al., 2017). Furthermore, based on recent extensive molecular analysis of the phylogenetic relationships of extant Asteroidea (Linchangco et al., 2017), the occurrence of PPLNP1‐type (SMP‐type) and PPLNP2‐type proteins in *A. rubens* (order Forcipulatida) and in *A. planci* (order Valvatida) suggests that both of these PP/OK‐type precursors would have been present in the common ancestor of all extant starfish. Previous studies have shown that PPLN1‐type (SMP‐type) peptides act as muscle relaxants in *P. pectinifera* (order Valvatida and in *A. rubens* (order Forcipulatida) and therefore it seems likely, based on asteroid phylogeny (Linchangco et al., 2017), that PPLN1‐type peptides act as muscle relaxants in all extant starfish. This study is the first to reveal the myorelaxant activity of PPLN2‐type peptides in a starfish species—*A. rubens* (order Forcipulatida). It will be interesting, therefore, to investigate if PPLN2‐type peptides also act as muscle relaxants in starfish that belong to other starfish orders—for example, in *P. pectinifera* and/or *A. planci*, both of which are species belonging to the order Valvatida. Looking beyond starfish, it would be of interest to determine if PP/OK‐type neuropeptides act as muscle relaxants in other echinoderms Addressing this issue is feasible because precursors of PP/OK‐type neuropeptides have been identified in other echinoderms, including two precursors in the sea urchin *S. purpuratus* (SpPPLNP1 and SpPPLNP2; Rowe & Elphick, 2012), one precursor in the sea cucumber *A. japonicus* (Rowe, Achhala, & Elphick, 2014) and one or more precursors in several brittle star species (Zandawala et al., 2017).

As discussed in detail previously (Lin, Egertová et al., 2017), comparative analysis of the physiological roles of PP/OK‐type neuropeptides in the Bilateria is limited in as much as the echinoderms are the only deuterostomian phylum in which PP/OK‐type neuropeptides have been identified. Thus, the functional characterization of PP/OK‐type neuropeptides in starfish reported here and in two previous articles (Kim et al., 2016; Lin, Egertová et al., 2017) represents the totality of our knowledge of physiological roles of PP/OK‐type neuropeptides in deuterostomes. This contrasts with a much more extensive series of studies on PP‐type neuropeptides in mollusks and OK‐type neuropeptides in arthropods. It is clear from these studies that PP/OK‐type neuropeptides have a wide variety of physiological roles in protostomes, including regulation of muscle and ciliary activity associated with locomotor activity in mollusks (Hall & Lloyd, 1990) and regulation of both the frequency and amplitude of hind‐gut contraction in arthropods (Stangier et al., 1992). A unifying theme among the pharmacological actions of PP/OK‐type neuropeptides in protostomes is stimulatory effects, with the effector tissues often being muscular in nature. Thus, this contrasts with the relaxing effects of PP/OK‐type neuropeptides on starfish muscle reported here and previously (Kim et al., 2016; Lin, Egertová et al., 2017). We have speculated that this may reflect an excitatory–inhibitory transition in the roles of PP/OK‐type neuropeptides as regulators of muscle activity that accompanied the divergence of protostomes and deuterostomes (Lin, Egertová et al., 2017). To address this issue, investigation of the occurrence and physiological roles of PP/OK‐type neuropeptides in a variety of deuterostomian and protostomian taxa is now needed. Furthermore, discovery of the receptors that mediate the effects of PP/OK‐type neuropeptides will be necessary to gain a deeper understanding of the evolution PP/OK‐type neuropeptide signaling in the Bilateria.

## CONFLICT OF INTEREST

The authors declare that they have no conflict of interest.
